# On Nature’s Strategy for Assigning Genetic Code Multiplicity

**DOI:** 10.1371/journal.pone.0148174

**Published:** 2016-02-05

**Authors:** Simone Gardini, Sara Cheli, Silvia Baroni, Gabriele Di Lascio, Guido Mangiavacchi, Nicholas Micheletti, Carmen Luigia Monaco, Lorenzo Savini, Davide Alocci, Stefano Mangani, Neri Niccolai

**Affiliations:** Department of Biotechnology, Chemistry and Pharmacy, University of Siena, Siena, Italy; Inserm U869, FRANCE

## Abstract

Genetic code redundancy would yield, on the average, the assignment of three codons for each of the natural amino acids. The fact that this number is observed only for incorporating Ile and to stop RNA translation still waits for an overall explanation. Through a Structural Bioinformatics approach, the wealth of information stored in the Protein Data Bank has been used here to look for unambiguous clues to decipher the rationale of standard genetic code (SGC) in assigning from one to six different codons for amino acid translation. Leu and Arg, both protected from translational errors by six codons, offer the clearest clue by appearing as the most abundant amino acids in protein-protein and protein-nucleic acid interfaces. Other SGC hidden messages have been sought by analyzing, in a protein structure framework, the roles of over- and under-protected amino acids.

## Introduction

Soon after *Escherichia coli* genetic code was deciphered [1] and found to be almost universal [2], many hypotheses have been proposed to explain how the standard genetic code (SGC) evolved among the huge number of possible alternatives [3–9]. Indeed, the limited number of SGC exceptions has been fully characterized [10] as well as species-specific biases in the use of SGC codon repertoire [11–12]. It is stably accepted that in SGC amino acid assignments have not been given randomly [13–15], being instead a product of selection [16–18], even though not fully optimized in order to allow for some evolutive freedom [19]. In spite of the extensive computational efforts which have been recently made, a consistent framework for explaining the overall rationale of codon multiplicity assignment [20–22] has not yet been found [15, 23].

SGC ensures the translation each of the naturally occurring amino acids and the translation stop message at very different extents. Indeed, the 64 different combinations of RNA nucleotides have been assigned so that a group of eleven amino acids, including Asn, Asp, Cys, Gln, Glu, His, Lys, Met, Phe Trp and Tyr, are protected from translation errors with a number of codons below the average value of three. The assignment of three codons to Ile and stop translation message leaves fourteen possibilities to overprotect the remaining eight amino acids. Among the remaining amino acids, Ala, Gly, Pro, Thr and Val got just one extra codon, allowing a six-codons benefit to Arg, Leu and Ser.

The observation that Leu, the most abundant amino acid in all the protein sequences deposited in UniProt databases [24], is also among the those which receive the highest protection by SGC, could suggest that occurrence in protein sequences is the basis for codon multiplicity. This hypothesis, already proposed in early discussions on SGC rationale [25], together with possibility that the SGC degeneracy determines amino acid frequency in proteins, is contradicted by the Arg case. Arg, indeed, exhibits at the same time average abundance in proteins and maximum SGC protection. Therefore, alternatives must be sought to find the origins of SGC biased codon assignment for incorporating all the amino acids that have survived Nature’s selection.

As suggested by the strict dependence of protein functions on tridimensional structures, it is mandatory to study what amino acids do in a specific structural environment for defining accurately their functional attitudes. Thus, the role of each amino acid in different inner or outer protein regions can be analyzed in detail, by manual inspection, yielding powerful information on specific biological process. However, automatic high throughput screening of structure databases can be differently informative, provided that sufficiently large repertoire of structural data can be taken into account, giving the unique opportunity to define general aspects of Biology at atomic resolution. This is nowadays possible by using the information contained in the Protein Data Bank, PDB [26], together with Structural Bioinformatics procedures. Indeed, screening of short interatomic distances in *ad hoc* subsets of PDB files can produce huge amount of data that can be analyzed and categorized in different ways, as it has been done in the present report.

## Material and Methods

We used Ensembl BioMart tool available from the URL: http://www.ensembl.org/biomart to determine the natural abundance of each nucleotide in human coding sequences. From a total of 93,493 human coding sequences, 105,159,508 nucleotides have been considered to calculate the occurrence of A, T, C and G, resulting to be 0.262, 0.219, 0.257 and 0.262 respectively. By multiplying the latter values for each codon of SGC and by summing over all the combinations given to natural amino acids, their expected frequency has been calculated. Human protein sequences have been used to calculate individual amino acid frequencies.

Amino acid contacts at the protein-protein and protein-nucleic acid interfaces, separately for each datasets, have been analyzed atom by atom with two EBI tools, PDBsum [27] and NUCPLOT [28], respectively. PDBsum and NUCPLOT contact profiles have been parsed with Python scripts in order to generate data plots like the ones shown in this report. By using NUCPLOT, close distance interactions between protein and nucleic acids atoms have been collected by using default maximum threshold values of 3.00 and 3.35 Ǻ for hydrogen bonds (HB), and hydrophobic contacts, respectively. In the case of PDBsum, default threshold values of 3.5 and 4.0 Ǻ were used to select close interatomic contacts at the protein-protein interface. Henceforth in this report, close interatomic contacts are meant under the limits defined above. Protein and nucleic acids atoms are always named according to the PDB nomenclature (description given at http://www.bmrb.wisc.edu/referenc/nomenclature). Depth for all the atoms of a subset of protein PDB structures (*vide infra*) has been evaluated by using SADIC (Simple Atom Depth Index Calculator) algorithm by using the freely downloadable software at http://www.sbl.unisi.it. The ratio between the exposed volume of a probing sphere of radius r_0_ centered on atom i, V_i_, and the exposed volume of the same sphere when centered on an isolated atom, V_0_, has been considered as a measure of atom depths defined as depth indexes, D_i_ [29]. Each protein residue has been labelled according to the maximum D_i_ value found along its side chain. Distribution in seven structural layers has been proposed to describe the natural amino acids content of inner and outer protein regions [30]. Open source PyMOL v. 1.7.1.0 has been used for molecular structure presentation and analysis.

## Results

**Fig 1:**
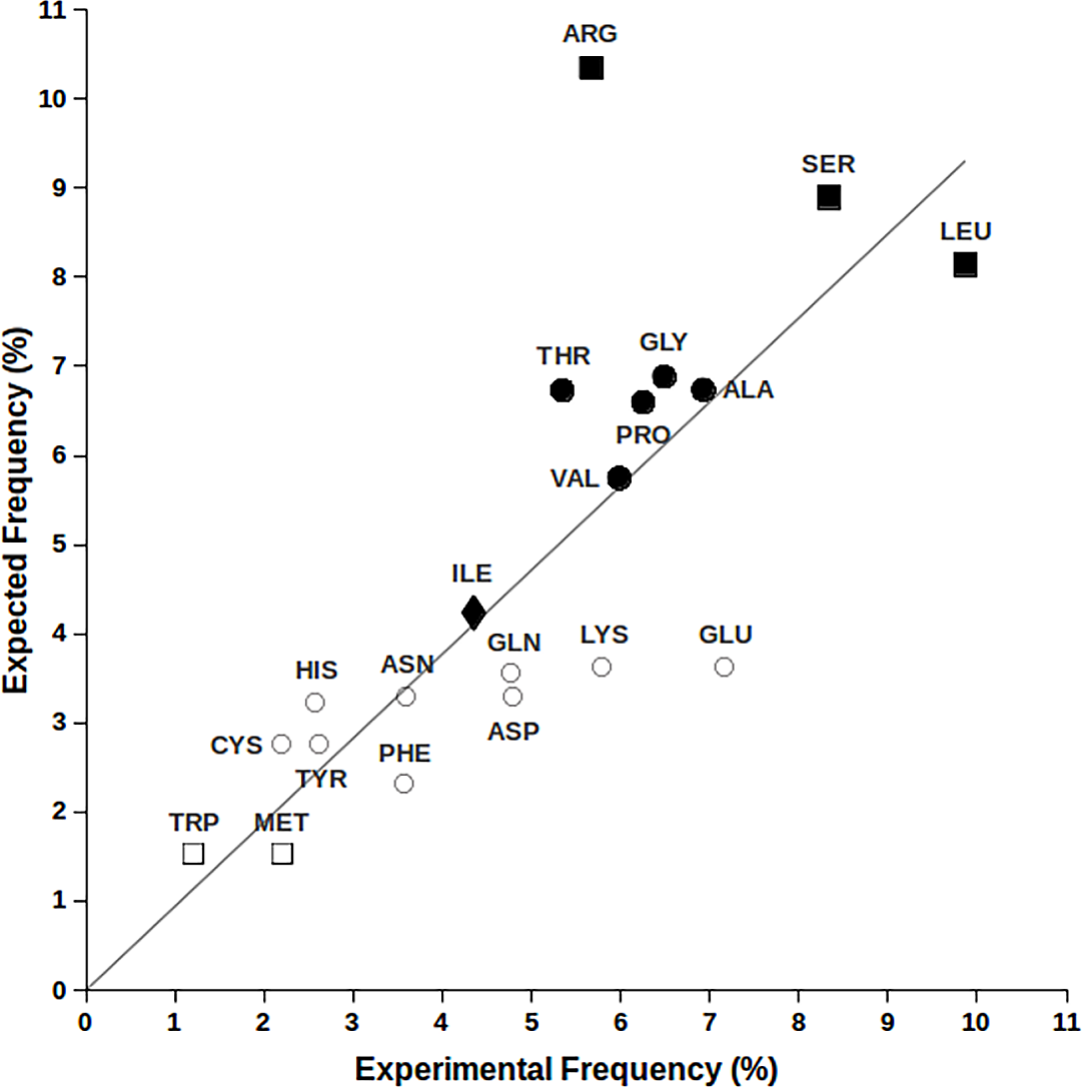
Expected vs experimental amino acid frequency in human protein sequences. Amino acids with 6, 4, 3, 2 and 1 codons are labelled respectively with ■, ●, ♦, ○ and □.

We explored the correlation between amino acid natural abundance and the corresponding number of competent codons by comparing expected amino acid frequencies, obtained on a genomic basis, with the ones found in sequenced proteins. In order to take into account only homogeneous species-specific data, human genome has been chosen for this investigation assisted by Ensemble BioMart tool to derive nucleotide frequencies from the obtained coding sequences. From retrieved DNA sequences the occurrence of each nucleotide was determined (see Methods section) and A, C, G and T were respectively 26.20%, 25.68%, 26.23% and 21.90%. As reported in Fig 1, the sum of the expectation values for each codon of natural amino acids, as resulting from the product of their nucleotide occurrence, is compared to the amino acid frequency found in human protein sequences.

By inspection of Fig 1, it is possible to note that expected and observed amino acid frequencies exhibit a good correlation with a R2 = 0.91, supporting the initial assumption that number of codons and amino acid occurrence in proteins are strictly related [25]. However, Arg and Glu appear particularly distant from the theoretical curve, offering us the initial clue for unveiling biased pathways of SGC evolution.

The possibility that the number of alternative codons for each amino acid could have evolved to protect those exhibiting specific roles, has been taken into account by searching common features for the amino acids with six codons, i.e. Leu, Arg and Ser. The fact that Life, at atomic resolution, might be considered a complex sum of intermolecular interactions, prompted us to check first for the relevance of the latter three amino acids in protein-nucleic acids and protein-protein interactions.

The wealth of structural information freely available from the Protein Data Bank, PDB [26], allows high throughput analysis of PDB files for calculating amino acid occurrence at the interface of protein-nuclei acid and protein-protein complexes. As a preliminary step of our investigation, we assembled PDB derived datasets containing all the interfaces which are indicated by PISA analysis. Non-redundant structural datasets, derived from PDB files available on May 2015, contained 663, 279 and 10,960 structures respectively for protein-DNA, protein-RNA and protein dimers, by far the most abundant oligomeric state of protein-protein complexes in the PDB.

**Fig 2:**
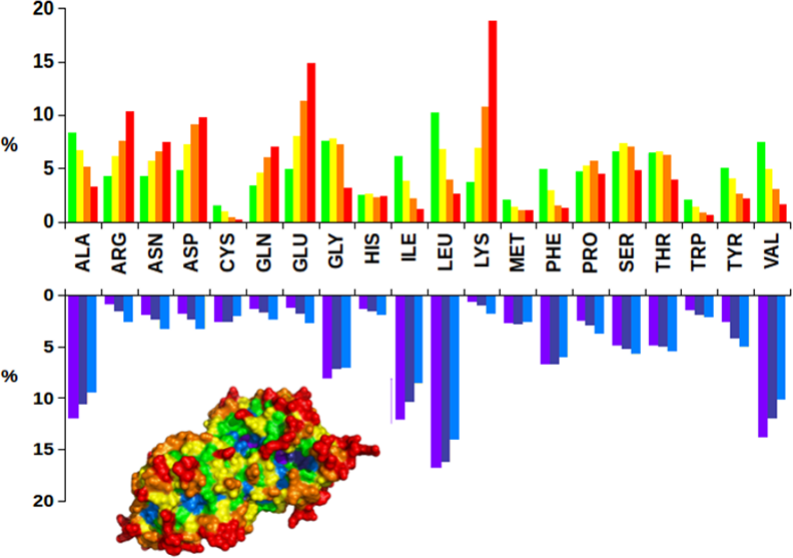
Amino acid distribution of DOOPS protein structural layers. Amino acid contents have been calculated for proteins selected on August 8, 2015 under the following limits: i) experimental method: X-RAY, ii) number of chains: 1, iii) oligomeric state: 1, iv) chain type: protein and v) number of entities: 1. For one of these proteins, PDB ID code 3NSM, amino acid composition of structural layers is shown in a PyMOL representation. Colors are given according to D_imax_ intervals: 0-<0.2 purple blue; 0.2-<0.4 deep blue; 0.4-<0.6 marine; 0.6-<0.8 green; 0.8<1.0 yellow; 1.0<1.2 orange; >1.2 red (PyMOL color nomenclature).

The analysis of the Structural Bioinformatics data, including amino acid compositions of protein-protein and protein-nucleic acid interfaces, is greatly facilitated by our original approach based on atom depth calculations [30]. Indeed, we have proposed a computational procedure to define protein structural layers where amino acids are located on the basis of their atom depths. This has been done for all proteins whose overall shape in the PDB crystal structures is considered minimally disturbed by interactions with other molecules. Thus, a Dataset Of Only Protein Singles, DOOPS [29], has been created by selecting only those proteins fulfilling the limiting conditions of PDB advanced search reported in Fig 2 caption and upon removal of redundant files.

It is important to note that frequency of natural amino acids in this PDB selection is almost identical to the one in whole UniProt databases [24], confirming the statistical significance of DOOPS proteins. We then performed atom depth calculation on each of the 2,158 proteins present in DOOPS by using SADIC algorithm [30] to derive amino acid depth. According to the procedure described elsewhere [29], the amino acid content of all the structural layers of DOOPS files has been quantified and results are reported in Fig 2.

As expected, polar and charged amino acids occupy predominantly the outer structural layers, Lys and Glu having, by far, the highest occurrences. The amino acid composition profile of outer structural layers of DOOPS proteins can be considered as a reference for comparing characteristic patterns of amino acid occupancy of protein-nucleic acids and protein-protein interfaces.

**Fig 3:**
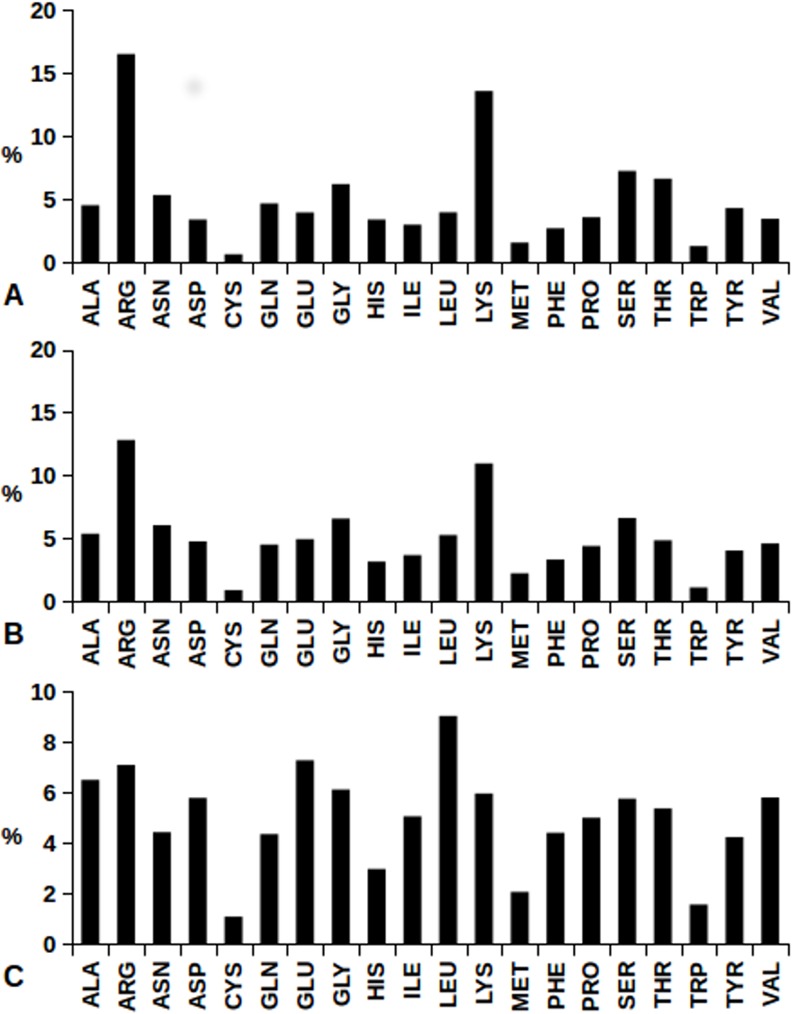
Interatomic close contacts at protein-protein and protein-nucleic acid interfaces. Percent histograms refer to frequencies of amino acid involvement from (A) 55,149 protein-DNA contacts, (B) 26,573 protein-RNA contacts and (C) 1,177,192 dimer protein-protein contacts which have been found in our datasets.

Systematic search of amino acids involved in protein-protein and protein-nucleic acid interactions has been performed on the basis of PDB files indicated by PISA [31] to contain interfaces between i) protein and DNA, ii) protein and RNA, iii) protein-protein in dimers. Uniprot databases [24] have been used to remove protein redundancy from all the datasets obtained by PISA. The amino acid compositions of protein-RNA and protein-DNA interfaces from all the structures of our datasets are shown in Fig 3A and 3B: the largest amino acid occurrence is exhibited by Arg, particularly at the protein-RNA interface, followed by Lys.

This finding is consistent with the fact that Arg and Lys, with their positive side chains are the best candidates to interact with the negative charges which are distributed along DNA and RNA backbones.

**Fig 4:**
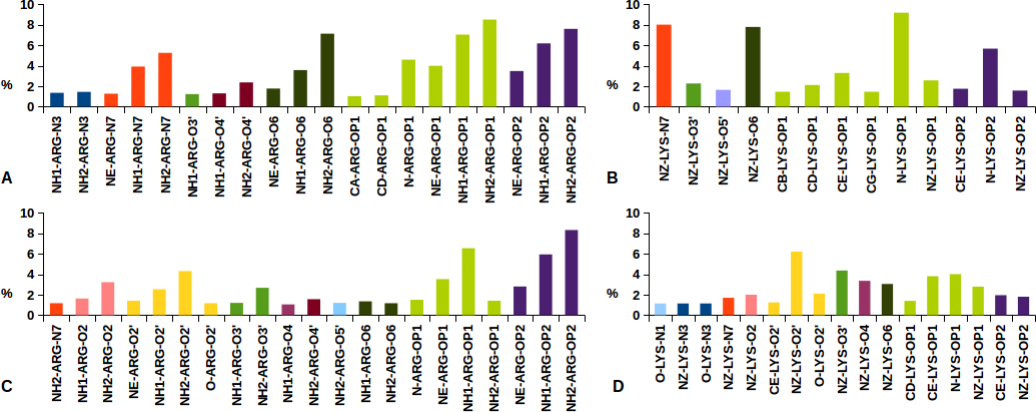
Protein close contacts with nucleic acids involving Arg and Lys atoms. Histograms report the number of Arg and Lys interatomic contacts in DNA (A, B) and RNA (C, D) interfaces occurring most frequently than 1%. Different histogram colors refer to the same nucleic acid atoms.

For a detailed structural analysis of protein-nuclei acid interactions, the EBI tool NUCPLOT [27] has been used for all the structures of our datasets. Fig 4 summarizes the most frequent Arg and Lys interactions with DNA and RNA atoms delineating some interesting features.

It is apparent how Arg NH1 and NH2 atoms are predominantly involved in approaching DNA and RNA backbones, through hydrogen bonding to OP1 and OP2 oxygen atoms, see Fig 4A and 4C. From Fig 4A it is interesting to note that similar Arg NH1/NH2 interactions with DNA O6 and N7 HB acceptors also occur. The same feature is not observed in Fig 4C, as in all the Arg-RNA interactions outside the NH1/NH2-OP1/OP2 network are below 4% of the total ones. Fig 4B and 4D highlight that Lys backbone amide approaches DNA and RNA OP1 and OP2 oxygens more frequently than NZ, leaving to the lysyl amino group the chance to interact with other HB acceptors from nucleic acid backbones or nucleobases. Consequently, as far as the protein-DNA/RNA interaction is concerned, different behaviors emerge for the two amino acids which are more frequently found at the protein-nucleic acid interface: i) the sticky Arg side chain interacts mainly with OP1 and OP2 oxygens and ii) Lys is involved in nucleic acid backbone interactions mainly through its amide group, leaving to the amino side chain the freedom to bind to the nucleobase HB acceptors.

**Fig 5:**
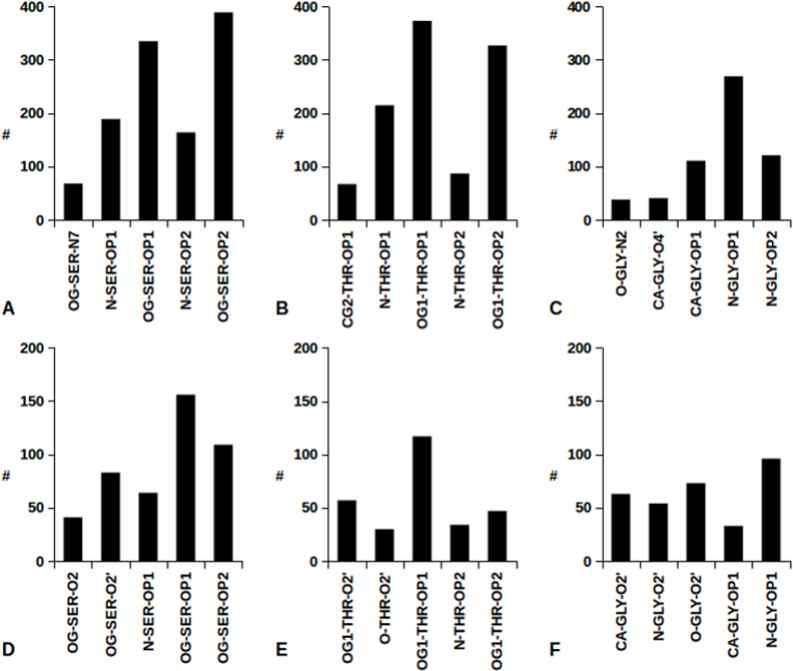
Protein close contacts with nucleic acids involving Ser, Thr and Gly atoms. Histograms report the number of the five most frequent interatomic contacts of Ser, Thr and Gly in DNA (A-C) and RNA (D-F) interfaces.

Fig 3 shows the abundance, always above the average, of Ser and Thr at protein-nucleic acid interface. The NUCPLOT analysis for the latter two amino acids shows HB formation, at similar extents, between their hydroxyl moieties and the OP1/OP2 acceptors of both DNA and RNA backbones. Therefore, due to the absence of bulky side chains in Ser and Thr, this interaction favors backbone to backbone protein-nucleic acid close approaches. A similar behavior is observed also in the case of Gly, as the corresponding backbone amide hydrogen is frequently involved in HB with the OP1/OP2 oxygens of nucleic acid backbone, see Fig 5.

**Fig 6:**
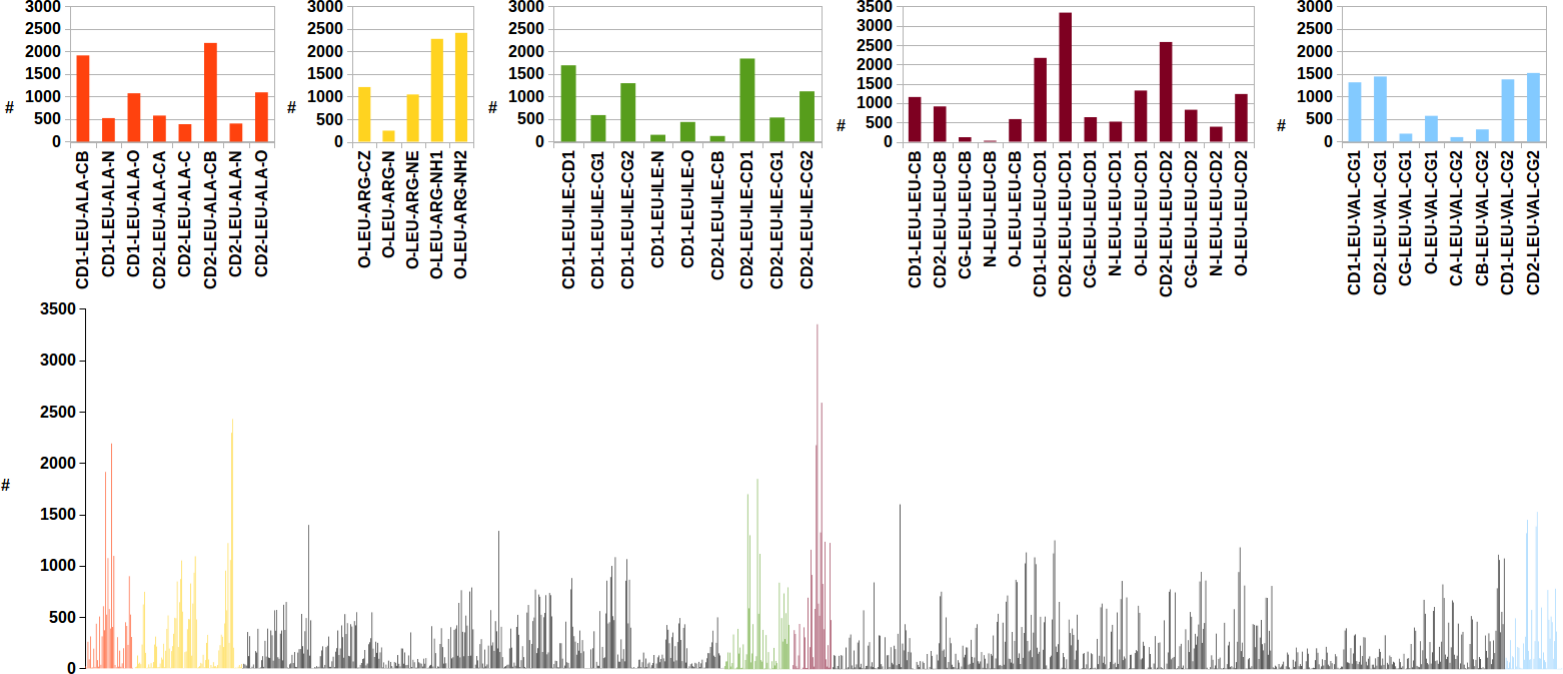
Leu close contacts with neighboring amino acids at the dimer protein-protein interfaces. Red, yellow, green, magenta and cyan histograms refer to the number of contacts between Leu atoms and the ones of Ala, Arg, Ile, Leu and Val respectively. In the insets, details for the most populated histograms are given.

Amino acid occurrence in dimeric protein-protein interfaces of our data set has been analyzed. Fig 3C shows percent amino acid frequencies at the protein-protein interface, indicating the large predominance of Leu, followed by Arg and Glu. In order to understand in more detail the role of natural amino acids in protein-protein interactions, another powerful EBI tool, PDBsum [26], has been used. It is apparent how protein-protein interaction landscape is much more complex than the ones discussed above for protein-nucleic acids. In the example given in Fig 6, close contacts of Leu atoms with the neighboring amino acids at protein-protein interface are shown. It is worth noting that Leu results as the most frequent Leu neighbor, followed by Arg, Ala, Ile and Val.

## Discussion

The Protein Data Bank, year after year, is hosting an increasingly large amount of structural data which are, individually, extremely precious for understanding biological mechanisms at atomic resolution. This data bank is now large enough to make possible Structural Bioinformatics approaches and to go further, as high throughput analyses of suitable collections of PDB files may yield new information on basic aspects of Biology. Thus, for a sub-set of PDB proteins having overall tertiary structures which are minimally influenced by intermolecular interactions, amino acid composition of inner and outer structural layers have been defined, see Fig 2 for an update of previously published data [30].

**Fig 7:**
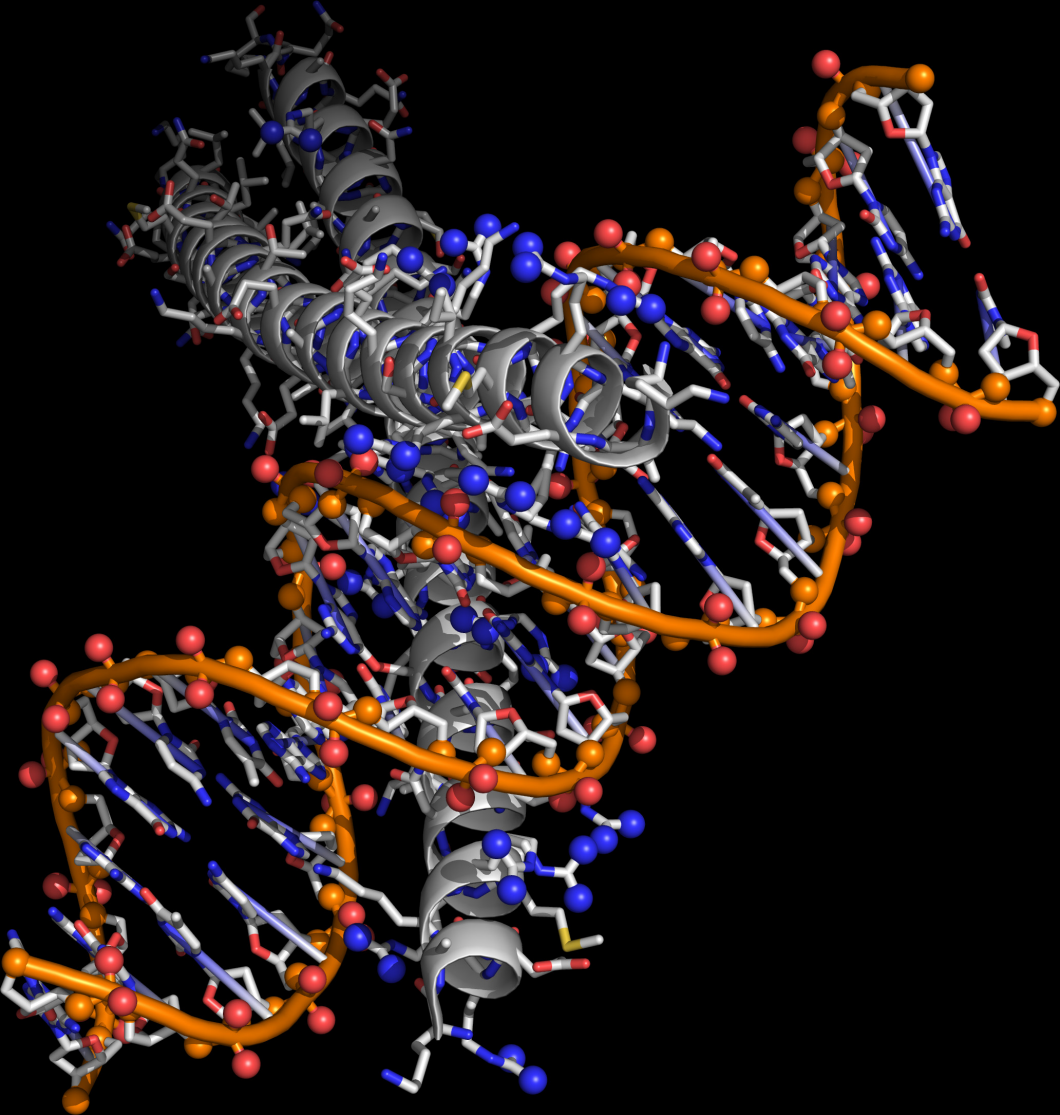
The interaction between a Leu zipper protein and a DNA duplex fragment. Arg side chain NH1 and NH2 atoms are shown as blue spheres. OP1-OP2 and O3’-O5’ DNA backbone atoms are shown respectively as red and orange spheres (image generated by PyMOL with PDB ID: 2H7H).

In the present study, we performed high throughput screening of protein-protein and protein-nucleic acid interfaces on selected collections of PDB structures. It is of primary relevance the observation that amino acid composition profiles of protein surface are very different depending on the involvement in interactions with nucleic acids, with other proteins (Fig 3), or just with solvent molecules (Fig 2). Arg, indeed, appears as the most abundant amino acid at protein-nucleic acid interfaces, with a primary role of the guanidinium group in the binding of DNA and RNA backbones (Fig 4 and Fig 7).

Furthermore, Arg results to be second only to Leu in occupying protein-protein interfaces, in this case completing its mission of connecting biopolymers with the formation of salt bridges with Glu and Asp side chains. By assigning six alternative codons to minimize possible Arg translation errors, SGC clearly provides maximum protection to protein-protein and protein-nucleic acids interaction events.

The origin of Leu generous six-codons assignment, is not only due to its abundance in inner protein structural layers, see Fig 2, but also to the Leu highly frequent occurrence at protein-protein interfaces (Fig 3C).

The Leu atomic interaction profile shown in Fig 6, shows how Leu methyl groups contribute to dimer protein assembly in a way which is not confined to specific structural determinants, such as leucine zipper motifs [32]. The high relevance of hydrophobic effects in stabilizing protein-protein interactions is confirmed by the very frequent methyl-methyl contacts involving Ale, Ile and Val side chains. It is also interesting to note that, besides the huge number of the interatomic contacts defined by PDBsum at protein dimer interfaces, the abundance of Leu and Arg can be attributed also to the many close distance approaches between Leu carbonyl and Arg NH1 or NH2.

The fact that fourteen different codons are collectively assigned to Ser, Thr and Gly can be related to the already discussed relevance to yield anchoring effects between nucleic acid and protein backbones, and also to the observed frequent occurrence of the latter three amino acids which is well above the average (Fig 3C). The small size of Ser, Thr and Gly side chains can favor backbone to backbone proximity, allowing many side chain-side chain or side-chain-backbone interactions involving other amino acids.

To explain why Lys, abundant at protein-nucleic acid interfaces, has only two codons is not straightforward. However, it must be noted that all amino acid with charged side chains, apart from Arg, have just two codons. This feature leads to a marked SGC under-protection for Lys and Glu that exhibit the lowest ratios of the corresponding expected vs. experimental frequencies (Fig 1). Fig 2 reveals the answer to this problem by highlighting the common behavior exhibited by Glu and Lys: they are predominantly located in protruding surface regions, *i*.*e*. outer structural layers. It follows that any possible point mutations involving solvent exposed Lys and Glu has no effect on the protein folding process, contributing to reduced SGC protection from protein translation errors. It is rather obvious, indeed, that in the absence of correct protein folding, no proper interactions can occur. Indeed, SGC evolved to maximize the chances of correct protein folding as evidenced by the fact that all the aliphatic amino acids, the most frequently present in the inner protein structural layers, have a minimum of three codons.

Assigning only one codon to Met, the start signal for RNA translation, when three different options are given to terminate the same process, reveals another point of SGC rationale: after assuring proper protein interactions with nucleic acids and other proteins, after controlling correct protein folding, no special protection is given to protein production. In other words, it is better not to have a protein at all than having it not in the proper conformation for interacting as required.

The Structural Bioinformatics survey that we have carried out to unveil SGC criteria for codon multiplicity assignment to natural amino acids, clearly shows the reasons of Arg, Leu and Ser protection from translation errors:

i) Arg, with its sticky side chain, is the most used amino acids by Nature to stabilize protein-nucleic acids and, to a lesser extent, protein-protein interactions. ii) Leu, the most “popular” amino acid, acts mainly to stabilize the interior of proteins, but its presence on the surface is most frequently required for protein-protein docking through methyl-methyl interactions. iii) Ser, Thr and Gly are frequently found at the protein-protein and protein-nucleic acid interface allowing backbone to backbone short distance approaches.

As a final remark we want to underline that a huge amount of information is buried in the data generated in this study and specific details will be discussed in future reports. We like also to note that only Structural Bioinformatics procedures could reveal Nature's general trends for optimal protection of protein folding and interactions with other proteins and nuclei acids.

## Supporting Information

S1 Table The entire set of amino acid contacts occurring at protein-protein and protein-nucleic acids interfaces discussed in part in this report. (ODS)

S2 Table The entire set of interatomic contacts occurring at protein-nucleic acids interfaces discussed in part in this report. (ODS)

S3 Table The entire set of interatomic contacts occurring at protein-protein interfaces discussed in part in this report. (ODS)
